# TriPOINT: a software tool to prioritize important genes in pathways and their non-coding regulators

**DOI:** 10.1093/bioinformatics/bty998

**Published:** 2018-12-19

**Authors:** Asa Thibodeau, Dong-Guk Shin

**Affiliations:** Department of Computer Science & Engineering, University of Connecticut, Storrs, CT, USA

## Abstract

**Summary:**

Current approaches for pathway analyses focus on representing gene expression levels on graph representations of pathways and conducting pathway enrichment among differentially expressed genes. However, gene expression levels by themselves do not reflect the overall picture as non-coding factors play an important role to regulate gene expression. To incorporate these non-coding factors into pathway analyses and to systematically prioritize genes in a pathway we introduce a new software: Triangulation of Perturbation Origins and Identification of Non-Coding Targets. Triangulation of Perturbation Origins and Identification of Non-Coding Targets is a pathway analysis tool, implemented in Java that identifies the significance of a gene under a condition (e.g. a disease phenotype) by studying graph representations of pathways, analyzing upstream and downstream gene interactions and integrating non-coding regions that may be regulating gene expression levels.

**Availability and implementation:**

The TriPOINT open source software is freely available at https://github.uconn.edu/ajt06004/TriPOINT under the GPL v3.0 license.

**Supplementary information:**

Supplementary data are available at *Bioinformatics* online.

## 1 Introduction

Pathway analyses are often utilized to identify pathways that are enriched in differential genes between conditions (i.e. cases versus controls) to gain a better understanding of the biological processes that are affected by the phenotype of interest (e.g. a disease). Methods for pathway analysis over the years have fallen into three categories (Khatri *et al.*, 2012): (i) over representation analyses which count the number of differentially expressed genes within a pathway (Huang *et al.*, 2009a, b), (ii) functional class scoring which calculates enrichment scores of pathway gene sets (Subramanian *et al.*, 2005, 2007) and (iii) pathway topology analyses where pathways are translated into directed graphs or networks to incorporate directionality and interaction types such as activation or inhibition (Bokanizad *et al.*, 2016; Martini *et al.*, 2013; Sebastián-León *et al.*, 2013; Tarca *et al.*, 2009; Vaske *et al.*, 2010; Zhao *et al.*, 2017). Only a few pathway analyses have integrated pathways with additional data (Calura *et al.*, 2014). These analyses can lead to the identification of pathways whose functions are affected as a result of a disruption in the processes, e.g. via a single nucleotide polymorphism that might be associated with a disease state. However, the majority of single nucleotide polymorphisms are located in non-coding regions (Hindorff *et al.*, 2009), where determining their phenotypic outcome is a challenging task. Moreover, non-coding regions include enhancers, which are *cis*-regulatory elements that have been shown to precisely regulate a gene’s expression in cell-specific contexts (Ong and Corces, 2011), further reinforcing the importance of incorporating non-coding information with gene expression and pathway analyses. In recent years, several assays have been developed, including ChIA-PET (Fullwood *et al.*, 2009), HiC (Lieberman-Aiden *et al.*, 2009) and HiChIP (Mumbach *et al.*, 2016), to identify chromatin loops that bring non-coding regions in close proximity of their target genes’ promoters, which help uncover their phenotypic outcome. Furthermore, we recently showed that the degree to which a gene interacts with non-coding regulators has been associated with its importance in the studied cell type (Thibodeau *et al.*, 2017), which can be used to further prioritize non-coding regions and their targets for experimental validation. As more data and methods become available for linking non-coding regions to their target genes, it becomes increasingly important to provide the computational tools to incorporate non-coding regions into downstream analyses of differentially expressed genes and pathways.

**Fig. 1.**
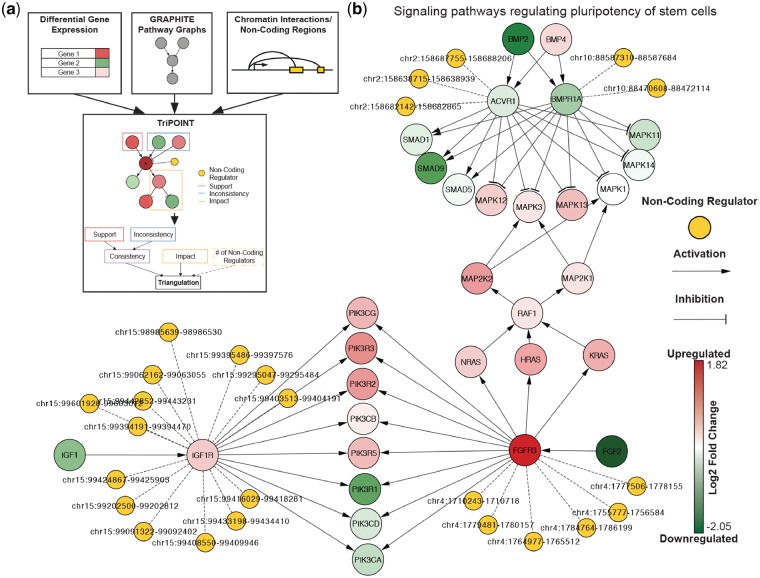
(**a**) Schematic of TriPOINT. Differential gene expression, pathways from GRAPHITE and chromatin interaction data and/or non-coding regulator locations are integrated into TriPOINT to identify perturbed genes/pathways and non-coding regulators. *Triangulation* scores are obtained by combining *consistency*, *impact* and the number of non-coding regulators targeting the gene. (**b**) Sub-graph of the Signaling pathways regulating pluripotency of stem cells pathways for early stage breast cancer patients older than 50 from TCGA. Non-coding regulators are shown from the genes with significant *triangulation* scores in the pathway: ACVR1, BMPR1A, IGF1R and FGFR3

Current approaches for pathway analyses are restricted to genes (Bokanizad *et al.*, 2016; Calura *et al.*, 2014; Huang *et al.*, 2009a, b; Martini *et al.*, 2013; Sebastián-León *et al.*, 2013; Subramanian *et al.*, 2005, 2007; Tarca *et al.*, 2009; Vaske *et al.*, 2010; Zhao *et al.*, 2017) but do not incorporate non-coding regulatory elements. To fill this gap we developed Triangulation of Perturbation Origins and Identification of Non-Coding Targets (TriPOINT) (Fig. 1a), software designed to identify genes perturbed in pathways and non-coding regulatory elements regulating them. TriPOINT offers a novel method for pathway analysis by identifying the genes that are the most affected under a condition by using multiple novel scoring metrics to uncover the impact of a gene’s perturbation on the network and by providing the ability to integrate these genes with non-coding regions using chromatin interaction datasets. TriPOINT is an easy to use and flexible tool for furthering existing methods for pathway analyses, which can lead to the identification of not only the most relevant genes for a phenotype but also their non-coding regulators.

## 2 Materials and methods

TriPOINT is implemented in Java, incorporating pathway graphs from the GRAPHITE (Sales *et al.*, 2012) R package through RServe (Urbanek, 2003; ISSN 1609-395X). Methods from our software QuIN (Thibodeau *et al.*, 2016) are utilized to integrate chromatin interaction data to identify non-coding regulators. Finally, the Cytoscape (Shannon, 2003) java application is used as a platform for visualization of TriPOINT JSON files which are easily imported and display pathways augmented with differential expression values and non-coding information (see Fig. 1b for an example).

### 2.1 Ranking of genes in pathways in terms of their significance

TriPOINT utilizes graph representations of pathways obtained from GRAPHITE (Sales *et al.*, 2012) to analyze the expression of genes in pathways using our novel *triangulation* measure based on four basic metrics: *inconsistency*, *support*, *consistency* and *impact* (Supplementary Material). Expression values of immediate upstream genes are scored using *support* and *inconsistency* measures to quantify how much a gene’s expression is ‘supporting’ or going against the pathway’s activation or inhibition status (Supplementary Fig. S1). These two metrics are combined to define the *consistency* score where negative values reflect perturbed genes and positive values reflect genes following the expected expression pattern in the pathway. The *impact* score quantifies the downstream effect of a gene’s expression. Breadth first search is employed to identify the sub-graph of downstream genes that support their upstream activation/inhibition interactions where each downstream gene’s expression is normalized using exponential decay as a function of the graph edge distance from the source gene. *Consistency*, *impact* and optionally the number of non-coding regulators of the gene are combined to calculate the *triangulation* score, which is used to identify perturbed genes with the highest downstream impact and optionally ones with more interactions with non-coding regulators, which may influence their activity within the pathway. Our triangulation score maintains the sign of the consistency score so it can be used to identify both (i) gene inconsistent with their upstream targets and (ii) genes that are highly supported by their upstream associated genes, which can be useful for identifying enriched pathways. To assess the significance of each score, TriPOINT calculates permuted *P*-values. Permuted *P*-values are obtained by randomly reassigning gene expression values between genes from the expression values provided and recalculating scores based on the number of permutations to generate a null distribution.

TriPOINT is currently available for Human pathways and designed to be used with differential expression data. Although differential expression data are the preferred metric, other metrics for gene expression may be applied with appropriate parameter configurations.

### 2.2 Integration with non-coding regulators

We implemented two approaches to incorporate non-coding regulators into the graph representations defining pathways. The first approach utilizes chromatin interaction loops from genome-wide assays such as ChIA-PET (Fullwood *et al.*, 2009), HiC (Lieberman-Aiden *et al.*, 2009) or HiChIP (Mumbach *et al.*, 2016) datasets. Methods available from our software QuIN (Thibodeau *et al.*, 2016) were employed to construct a chromatin interaction network to identify loci directly interacting with genes in a pathway. If chromatin interaction data are not yet available for the given cell type, TriPOINT attempts to identify non-coding regulators based on proximity, assigning non-coding regions provided by the user to genes within a user-defined distance from the transcription start site. *P*-values relating to the significance of the number of non-coding regulators targeting a gene are calculated based on the Poisson distribution (Supplementary Material).

## 3 Results

We demonstrate the efficacy of TriPOINT in a case study by analyzing all stage-one breast cancer RNA-seq samples from females older than 50 (*n* = 7) profiled by The Cancer Genome Atlas (Koboldt *et al.*, 2012), which we obtained through the National Cancer Institute Genomic Data Commons (Grossman *et al.*, 2016) portal. We identified differentially expressed genes using all individuals by comparing their tumor and normal samples using DESeq2 (Love *et al.*, 2014). We employed TriPOINT on the differential expression data using KEGG (Kanehisa and Goto, 2000; Kanehisa *et al.*, 2016, 2017) pathway graphs available from GRAPHITE (Sales *et al.*, 2012). Non-coding regulators were included in these graphs by integrating MCF-7 (an early stage breast cancer cell line) DNASE-Seq (GSE32970) and RNA-Pol2 ChIA-PET (GSE39495) datasets from ENCODE (Dunham *et al.*, 2012). Genes/pathways were then selected using *triangulation* scores.

We obtained 864 gene/pathway combinations with *triangulation* score *P*-value <0.005. We observed 682 gene/pathway combinations with positive triangulation scores, 90 of which were in cancer pathways. Positive *triangulation* scores represent those gene/pathway combinations that are consistent with their respective surrounding pathway topology. We focused on the 182 gene/pathway combinations with negative *triangulation* scores as these represent genes that are perturbed in their respective pathways (i.e. they are inconsistent with the upstream genes activating or inhibiting them). Among these genes/pathways with negative *triangulation* scores, we identified ACVR1 and BMPR1A in the ‘Signaling pathways regulating pluripotency of stem cells’ KEGG pathway as among the top genes/pathways using these scoring criteria (Fig. 1b). We also noted other genes in the same pathway, namely FGFR3 and IGF1R with significant *triangulation* scores. ACVR1 and BMPR1A have each been previously associated with breast cancer (Slattery *et al.*, 2013). Interestingly, overexpression of FGFR3 and IGF1R in breast cancer (more specifically in MCF-7 for FGFR3) has been observed in previous studies (Farabaugh *et al.*, 2015; Fillmore *et al.*, 2010), each related to breast cancer expansion through stem cells. Further inspection of these genes revealed that many of the non-coding regulators interacting with IGF1R were also identified within two super enhancers (Hnisz *et al.*, 2013; Whyte *et al.*, 2013) in MCF-7: chr15: 99286560–99323022 and chr15: 99385754–99447217, revealing additional evidence that these loci are possibly in control of the IGF1R’s expression and merits further experimental study. This case study demonstrates the usefulness of TriPOINT in connecting non-coding factors to pathway analyses and prioritizing genes in pathways, bringing closer a more complete picture of underlying mechanisms in the control of expression by uncovering potential therapeutic targets via data integration. 

*Conflict of Interest*: none declared.

## Supplementary Material

bty998_Supplementary_Information
