# Supplementary material for: TriPOINT: a software tool to prioritize important genes in pathways and their non-coding regulators
Source: Bioinformatics. 2018 Dec 19;35(15):2686–9. doi: 10.1093/bioinformatics/bty998 (PMC6662310; doi:10.1093/bioinformatics/bty998)
Supplement: bty998_Supplementary_Information [file bty998_supplementary_information.pdf]

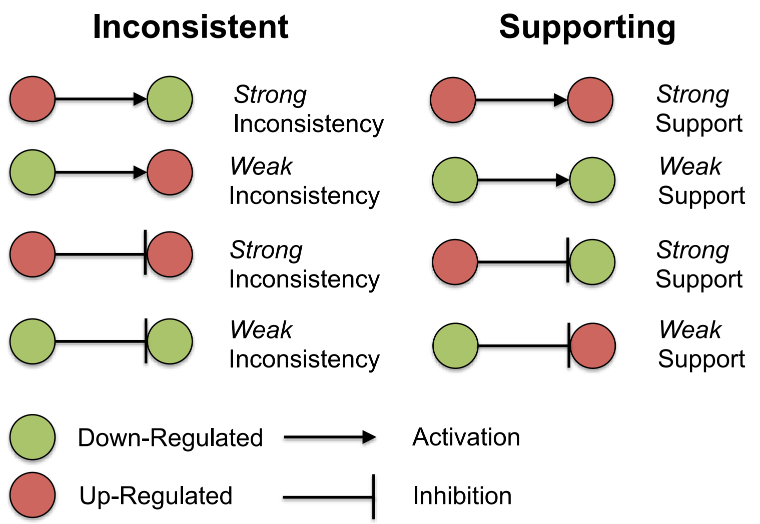

Supplementary Figure 1. Schematic of pathway interactions highlighting interactions that are inconsistent (going against activation/inhibition) and supporting (following activation/inhibition) where activation implies a gene is up-regulated and inhibition implies a gene is down-regulated as a result of its upstream gene being up-regulated. "Weak" annotations are introduced to separate complementary cases where upstream genes are down-regulated and may factor differently in their target's expression.

Definitions:

g – The collection of all genes found in the pathways.

g_i – One gene in the collection of genes.

p – The collection of all pathways found in the pathway database.

p_m – One pathway in the collection of pathways.

$\Delta \mathrm{Exp}(g_i)$ - Change in gene expression from control vs. treatment expression for gene g_i.

ρ - Parameter controlling how much influence gene expression/score has in score calculation where a values 1 or greater increase its influence and values less than 1 reduce the influence of gene expression/score. A value of 0 will eliminate the gene expression/score completely from calculation and TriPOINT will rely on the following thresholds for determining whether a gene is active/upregulated or inactive/downregulated only.

T_u - Active/upregulated gene threshold that specifies at which gene expression/score the gene will be considered active/upregulated. These must be set according to the scores provided such that the threshold for which genes should be considered activated/upregulated is set to this value using this parameter. For

example, values greater than a small positive number (i.e., 0.05) should be used when using log2 fold change data.

T_d - Inactive/downregulated gene threshold that specifies at which gene expression/score the gene will be considered inactive/downregulated. These must be set according to the scores provided such that the threshold for which genes should be considered inactivated/downregulated is set to this value using this parameter. For example, values less than a small negative number (i.e., -0.05) should be used when using log2 fold change data.

w - Parameter controlling the influence of "weak" interactions (pathway interactions involving down-regulated upstream genes) as a percentage. For example, a value of 0.5 will reduce the influence of weak interactions compared to strong interactions by half.

$E_{type}(g_1, g_2, p_m)$ - Returns the type of edge between two genes g_1 and g_2 in the pathway p_m. (ACTIVATION, INHIBITION, or ASSOCIATION (ignored in score calculation))

$d(g_i, g_j, p_m)$ - The number of edges between g_i and g_j in the pathway p_m.

$Us(g_i, p_m)$ - The set of immediate (edge distance = 1) upstream genes of a gene g_i in pathway p_m.

$Ds(g_i, p_m)$ - The set of downstream genes (of any edge distance) of gene g in pathway p_m derived from the sub-graph defined such that all genes are consistent (i.e. expression reflects activation/inhibition) with respect to their upstream genes beginning from the source gene g_i.

r - Controls the rate of exponential decay in the impact score. A value of 0 will remove the exponential decay induced by the number of edges between the source gene and the gene to be contributed into the impact score while a value of 1 will exponentially increase the amount to be divided by a factor $e^{(\text{distance})}$.

Inconsistency Score:

$$\text{Inconsistency}(g_i, p_m) = \sum_{g_j \in Us(g_i, p_m)} i_s \,|\Delta \text{Exp}(g_j)|^{\rho} + w \sum_{g_j \in Us(g_i, p_m)} i_w \,|\Delta \text{Exp}(g_j)|^{\rho}$$

$$i_s = \begin{cases} 1 & \text{if } E_{type}(g_j, g_i, p_m) = \text{ACTIVATION} \quad \Delta \text{Exp}(g_j) > T_u \quad \Delta \text{Exp}(g_i) < T_d \\ 1 & \text{if } E_{type}(g_j, g_i, p_m) = \text{INHIBITION} \quad \Delta \text{Exp}(g_j) > T_u \quad \Delta \text{Exp}(g_i) > T_u \\ 0 & \text{otherwise} \end{cases}$$

$$i_w = \begin{cases} 1 & \text{if } E_{type}(g_j, g_i, p_m) = \text{ACTIVATION} \quad \Delta \text{Exp}(g_j) < T_d \quad \Delta \text{Exp}(g_i) > T_u \\ 1 & \text{if } E_{type}(g_j, g_i, p_m) = \text{INHIBITION} \quad \Delta \text{Exp}(g_j) < Td \quad \Delta \text{Exp}(g_i) < T_d \\ 0 & \text{otherwise} \end{cases}$$

Support Score:

$$\text{Support}(g_i, p_m) = \sum_{g_k \in Us(g_i, p_m)} s_s \,|\,\Delta\,\text{Exp}(g_k)\,|^{\rho} + w \sum_{g_k \in Us(g_i, p_m)} s_w \,|\,\Delta\,\text{Exp}(g_k)\,|^{\rho}$$

$$s_s = \begin{cases} 1 & \text{if } E_{type}(g_k, g_i, p_m) = \text{ACTIVATION} \;\; \Delta\,\text{Exp}(g_k) > T_u \;\; \Delta\,\text{Exp}(g_i) > T_u \\ 1 & \text{if } E_{type}(g_k, g_i, p_m) = \text{INHIBITION} \;\; \Delta\,\text{Exp}(g_k) > T_u \;\; \Delta\,\text{Exp}(g_i) < T_d \\ 0 & \text{otherwise} \end{cases}$$

$$Sw = \begin{cases} 1 & \text{if } E_{type}(g_k, g_i, p_m) = \text{ACTIVATION} \;\; \Delta\,\text{Exp}(g_k) < T_d \;\; \Delta\,\text{Exp}(g_i) < T_d \\ 1 & \text{if } E_{type}(g_k, g_i, p_m) = \text{INHIBITION} \;\; \Delta\,\text{Exp}(g_k) < Td \;\; \Delta\,\text{Exp}(g_i) > T_u \\ 0 & \text{otherwise} \end{cases}$$

Consistency Score:

$$\text{Consistency}(g_i, p_m) = \text{Support}(g_i, p_m) - \text{Inconsistency}(g_i, p_m)$$

Impact Score:

$$\text{Impact}(g_i, p_m) = \sum_{g_m \in Ds(g_i, p_m)} \frac{|\,\Delta\,\text{Exp}(g_m)\,|^{\rho}}{e^{r*d(g_i, g_m)}}$$

Triangulation Score (without Non-Coding):

If either consistency or impact scores are 0, the triangulation score is 0 otherwise the following equation is used to calculate triangulation:

$$\text{Triangulation}(g_i, p_m) = \text{sign}(\text{Consistency}(g_i, p_m)) * \left[\frac{\text{minmaxnorm}(\text{Consistency}(g_i, p_m)) + \text{minmaxnorm}(\text{Impact}(g_i, p_m))}{2}\right]$$

Triangulation Score (with Non-Coding):

If consistency, impact scores are 0, or if the number of non-coding regulators is 0, the triangulation score is 0 otherwise the following equation is used to calculate triangulation:

$$\begin{aligned} &\text{Triangulation}(g_i, p_m) \\ &= \text{sign}(\text{Consistency}(g_i, p_m)) * \left[\frac{\text{minmaxnorm}(\text{Consistency}(g_i, p_m)) + \text{minmaxnorm}(\text{Impact}(g_i, p_m)) + \text{minmaxnorm}(\text{noncoding}(g_i))}{3}\right] \end{aligned}$$

The minmaxnorm function is the min max normalization of the score based on all scores across all genes/pathways within the score category. The noncoding function is the number of noncoding regulators targeting gene g_i.

Triangulation score is composed of 2-3 main parts. First, the consistency score measure the degree to which the gene is following or going against genes regulating it as suggested in the pathway. Second, the impact score measures how much the gene influences its downstream gene targets. For example, if the gene being measured is upregulated, it will have a greater impact score if its downstream genes are following expression patterns indicative of supporting pathway interactions. Triangulation scores are set to 0 if any of the combined scores are 0 to eliminate scores driven entirely by one metric. Triangulation scores will be in the range -1 to 1 where a -1 triangulation score refers to a gene that is inconsistent with upstream factors where as a triangulation of 1 refers to a gene that is consistent with upstream factors.

Non-Coding Regulator P-Value Calculation

TriPOINT provides p-values for the number of non-coding regulators to measure this significance independent of other metrics (e.g., triangulation score), allowing for broader applicability of TriPOINT for those strictly interested in the number of non-coding regulators for genes. Here we assume the number of non-coding regulators follows a Poisson distribution with parameters λ and *k*. The probability of *k* non-coding regulators interacting with a gene is calculated as:

$$prob(k) = \frac{\lambda^k}{k!e^{\lambda}}$$

where λ is the average number of interactions across all genes (i.e., the estimated probability of observing an interaction for a gene). The p-value reported by TriPOINT is obtained by subtracting the cumulative probabilities up to the observed number of interactions k from 1.

FDR Adjusted P-Values

In addition to P-Values, TriPOINT provides FDR adjusted p-values (q-values) obtained from the p.adjust statistical function from R using the Benjamini, Hochberger procedure (“fdr” method parameter in R). These values in combination with the reported p-values will enable users to appropriately select gene/pathway combination that are significant as reported by the p-value while having an idea that a certain portion of these will be false positives using the q-value.
